# The COVID-19 Elective for Pediatric Residents: Learning About Systems-Based Practice During a Pandemic

**DOI:** 10.7759/cureus.13085

**Published:** 2021-02-02

**Authors:** Maya S Iyer, Charmaine B Lo, Daniel J Scherzer, Doug MacDowell, Nita Gupta, Ellen McManus, Claire Stewart, Seth W Linakis, Rachel Stanley

**Affiliations:** 1 Division of Emergency Medicine/Pediatric Emergency Medicine, Nationwide Children’s Hospital, Columbus, USA; 2 Pediatrics, The Ohio State University College of Medicine, Columbus, USA; 3 Division of Emergency Medicine, Nationwide Children’s Hospital, Columbus, USA; 4 Abigail Wexner Research Institute, Nationwide Children’s Hospital, Columbus, USA; 5 Division of Critical Care Medicine/Pediatric Critical Care Medicine, Nationwide Children’s Hospital, Columbus, USA

**Keywords:** emergency medicine - emergency critical care - disaster medicine, covid-19 pandemic, curriculum implementation, curriculum development and evaluation, pediatrics emergency, asynchronous learning, systems based practice, incident learning systems, hospital response

## Abstract

Background: The coronavirus disease 2019 (COVID-19) pandemic has prompted pediatric residency programs to adjust the delivery of educational curricula and to update content relevant to the pandemic.

Objective: In this descriptive paper, we present how we rapidly developed and implemented a COVID-19 pandemic elective for pediatric residents.

Methods: This curriculum was established at a single tertiary care children’s hospital in June 2020. We used the ADDIE (analysis, design, development, implementation, evaluation) framework to develop a two-week elective (30 hours) consisting of six flexibly scheduled modules. We administered post-elective surveys and exit interviews to solicit feedback to improve the elective and obtain effectiveness of our educational interventions.

Results: We developed an asynchronous online COVID-19 Elective for Pediatric Residents. The curriculum modules focus on pathophysiology of severe acute respiratory syndrome coronavirus 2 (SARS-CoV-2), the disaster management ecosystem, simulation of clinical care, mental health ramifications, and public health consequences. We also include six in-situ experiences (visits to a drive-through COVID-19 testing site, testing laboratory and local public health department, a simulation of a critically ill child, and meetings with emergency managers and social workers) to solidify learning and allow for further reflection.

To date, eight participants have taken the elective. All participants strongly agreed on a five-point Likert item survey that the elective enhanced their knowledge in current evidence-based literature for COVID-19, disaster preparedness, hospital response, management of the critically ill child, and mental and public health ramifications. All participants agreed this curriculum was relevant to and will change their practice.

Conclusions: We demonstrate how a COVID-19 elective for pediatric residents could be quickly developed and implemented. The pilot results show that pediatric trainees value asynchronous learning, supplemented by relevant in-situ experiences. Moreover, these results suggest that this curriculum provides needed disaster response and resiliency education for pediatric residents.

## Introduction

Medical education must adapt and restructure in response to disasters [1,2]. Pediatricians are infrequently frontline providers for such events and rarely receive training in disaster response and resiliency [3]. However, the coronavirus disease 2019 (COVID-19) pandemic has forced some pediatricians to care for patients outside the traditional pediatric age range and try to learn and incorporate these concepts rapidly in real-time [4]. The Accreditation Council for Graduate Medical Education (ACGME) for Pediatrics Residency Programs states, “Residents must demonstrate an awareness of and responsiveness to the larger context and system of health care…as well as the ability to call effectively on other resources to provide optimal health care” but does not directly address disaster response [5]. Recent literature suggests that “given the limited resources during the COVID-19 outbreak, trainees [must] learn creative problem-solving skills and gain a practical understanding of the relationships of value, cost and quality to medical care [6].” The concept of disaster preparedness, in the setting of general public concern as well as the current COVID-19 pandemic, is not directly addressed. This suggests that disaster training was not previously a priority for pediatricians and that there may be a lack of relevant education in pediatric residency programs.

Learning about disaster preparedness, response, and resiliency is crucial as pediatricians are assuming the care of both pediatric and non-pediatric patients [7]. Although some non-pediatric residency training programs and medical school curricula have focused on disaster medicine concepts, presently, there are no published pediatric COVID-19 pandemic curricula focusing on response and resiliency specifically for trainees [8-12]. These prior studies concentrate on understanding of incident command systems, mass casualty response, hospital disaster plans including surge capacity, and field triage. These topics are necessary but insufficient for understanding the ramifications and response to the COVID-19 pandemic.

To address this gap in disaster training, as well as the challenges of content delivery during a pandemic, we developed an asynchronous COVID-19 pandemic elective for pediatric residents. This was done in collaboration with the Assistant Secretary of Preparedness and Response (ASPR) Grant awarded to establish the Eastern Great Lakes Pediatric Consortium for Disaster Response as a Pediatric Disaster Care Center of Excellence. Our goal was to create an asynchronous, self-directed elective providing real-time exposure to hospital pandemic response plans, patient care experiences, as well as synthesis of current evidence-based literature on severe acute respiratory syndrome coronavirus 2 (SARS-CoV-2). This descriptive study presents the curricular elements and initial results of the elective.

## Materials and methods

Setting and participants

This study occurred at a single tertiary care children’s hospital with a large pediatric residency program of approximately 40 residents per year. The elective’s curriculum was rapidly developed in May 2020 and implemented in June 2020. The residency program made three formal announcements promoting the availability of this elective. Participation in this elective was completely voluntary. The pediatric residency program at our institution allots 80% of the residents’ schedules to required rotations, as such, they may only have time in their schedules for two to three electives a year. 

Interventions

We created and launched an asynchronous online pandemic elective, supplemented by in-situ experiences. Due to the sparsity of publicly available pediatric disaster curricula, our elective presents an adaptive example of concepts borrowed from emergency medicine education and reframed with a pediatric focus [8,13-14]. We used the ADDIE (analysis, design, development, implementation, evaluation) framework to inform our curriculum [15]. ADDIE is a systematic framework for creating effective instructional experiences targeting knowledge gaps. We specifically selected the ADDIE framework, not only because of its simplicity, but also because ADDIE models work best for curriculum geared towards producing specific learning outcomes and behavior changes, such as this curriculum. The ADDIE model has been used effectively in the military, health care, and education industries [16].

Analysis

Given ASPR’s request to rapidly develop and implement a curriculum, we conducted an informal needs assessment among our research team and pediatric trainees, including pediatric emergency medicine fellows, which confirmed disaster medicine and resiliency concepts are not emphasized in pediatric residency curricula. The curriculum development team consisted of the subspecialty pediatric emergency medicine and critical care physicians, a disaster manager, a pediatric emergency medicine fellow, an associate residency program director, and an epidemiologist. This team met weekly during the ADDIE process to review learning objectives, assessment methods, and learning outcomes.

The curriculum development team decided that all curricular components would focus on treating the pediatric patient both medically and psychosocially during a crisis. To elicit changes in knowledge and future behavior, we developed an online post-elective survey for participants. In addition, we administered in-person exit interviews to solicit feedback to improve the elective.

Design

We adapted the content for this elective from disaster curriculums available for medical students and emergency medicine residents [8,13-14]. In addition, we solicited input from a local physician disaster expert. The two-week elective (30 hours) consists of six modules addressing the needs assessment topics (Supplemental Content 1). Module 1 focuses on the pathophysiology of SARS-CoV2 in children and pregnant women. We included literature on pregnant women because upon delivery, their medical history is crucial to managing ill neonates. Module 2 introduces the disaster management ecosystem, resilience life-cycle components, and emergency manager’s roles. Module 3 highlights the importance of incorporating the resiliency framework into disaster preparedness plans. Module 4 rehearses safe personal protective equipment donning and doffing and increases familiarity with management of COVID-19 related Multi-system Inflammatory Syndrome (MIS-C). Module 5 addresses mental health ramifications of disasters on children, healthcare providers, and the community. Module 6 focuses on the public health consequences of global disease. 

Each module has specific objectives and includes multiple modes of content delivery, including videos, readings, lectures and podcasts. In addition, we include six in-situ experiences to solidify learning and allow for further reflection according to adult learning theories and principles [17,18]. These experiences are visits to a drive-through COVID-19 testing site, testing laboratory, and local public health department, along with high-fidelity simulation of a critically ill child, and meetings with emergency managers and social workers.

Development 

Our curricular content is in a secure-online platform called The Learning Center (TLC). TLC is Nationwide Children’s Hospital’s learning management system. This system is where employees will access their yearly required curriculum as well as sign up for various instructor led trainings. TLC can be accessed remotely, hosts multiple resources in a centralized location, allows for administration of evaluations, and tracks progress.

A pediatric chief resident and physician with disaster medicine expertise reviewed each module for face and content validity. The curriculum development team met weekly during the ADDIE process to review learning objectives, assessment methods, and learning outcomes. 

We developed online surveys examining the knowledge, attitudes, and future behavior changes of participants. We also developed an in person exit interview for participants to provide further feedback. The online survey and interview questions were piloted among an emergency medicine resident and a medicine-pediatrics resident. 

Results from these instruments are aggregated every three to four months and then the curriculum is updated on TLC based on recent literature and trainee feedback.

Implementation

Each resident had an orientation with the curriculum course director (MSI) and the curriculum administrator (DM) to go over the contents, expectations, deliverables/assignments and also how to use TLC. The curriculum went live in June 2020.

Outcomes measured

Throughout the course, pediatric residents completed knowledge-based tests and reflective writing assignments. After completion of all the modules, a one-on-one exit interview was conducted by the authors (DM, CBL). This information, in conjunction with a post-elective survey on changes in knowledge of disaster response and resiliency (Supplemental Content 2) were utilized in the evaluation framework for this elective. These instruments were internally developed but provides feedback on the elective in real time, allowing the authors to implement relevant changes in a timely manner.

Analysis of outcomes

For this small descriptive study, we analyzed survey data using counts and percentages, and conducted an in-depth thematic analysis of exit interview data [19].

IRB statement

This study was deemed exempt by the IRB at Nationwide Children’s Hospital. 

## Results

To date, eight pediatric residents participated in this elective and no resident declined the exit interview. Participating residents were randomly split equally between postgraduate year (PGY) 2 and PGY3. Half of the participants identified as female. All participants completed the online portions of the elective and submitted written assignments and certifications. In addition, all participants strongly agreed on a five-point Likert scale that the elective enhanced their medical knowledge on current evidence-based literature for COVID-19, disaster preparedness, hospital response, management of the critically ill child, mental and public health ramifications. Every participant strongly agreed that the in-situ activities reinforced their knowledge. Finally, all participants strongly agreed that the elective enhanced their knowledge overall about pandemics and was relevant to and will change their clinical practice. 

Three prominent themes emerged from the thematic analysis of exit interviews: the curriculum addressed core disaster medicine concepts, the curriculum provided practical knowledge on local resources to call upon during crises, and the multimodal learning allowed for flexibility in participating in educational activities. Table 1 presents illustrative quotes. 

**Table 1:** Exit Interview Responses on How Course Content Enhanced Learning

Theme	Selected Quote(s)
Addressed Knowledge Gaps on Core Disaster Medicine Topics	“As a Resident, and going through medical school, they don't discuss Emergency Preparedness much. We don't understand the depths and how complex it is. Speaking with [emergency preparedness] and going through the NCH structure was eye opening.”
	“I was in the dark, if (disaster resilience and how hospitals and public organizations respond to pandemics) was mentioned, it was briefly mentioned in PowerPoint that a guest speaker talked about. This is the first time that we talked about it. The FEMA courses were awesome - those were well structured and broke everything down. FEMA and emergency preparedness were some of the most valuable parts of the course.”
	“I didn't know a whole lot about what went into (disaster resilience and response) - we had one day in med school - I didn't really know who all was in charge of responding to disasters - how they plan ahead of time - I definitely think I know a lot more now than I did before.”
	“I think it’s really shocking that we don't get more education about this type of thing in general in residency. I think it’s important for us to know how the hospital responds to a pandemic and who/what goes into it. For example, the Emergency Coordinator told me that the Incident Commander for COVID-19 is a physician who wasn't aware of these things and some terminology before the pandemic. We don't get that sort of education and it is now more relevant than ever.”
Knowledge on Local Resources	“The highlight was the Public Health Department [visit]. I was frustrated with myself for how long it took me to go there. The Public Health Department is astounding. The best way to say it - is that it is all inclusive. They have so many resources. During the tour I saw so many people in the waiting room that are part of the patient base. I felt that I should have reached out sooner - to patients and families."
	"Parents' health is so important for the child's health - I could have directed them to the Public Health Department for resources. It is interesting how complex their system is - it works so well and is so embedded in the community. Once you get it, it’s like, wow, you guys do so much.”
	“I enjoyed talking to X from You Matter. How her practice has changed doing things remotely - along with coping skills she has been encouraging. (It was a) good experience.”
Multimodal Learning/Flexibility	“The resources that were presented were very valuable - the disaster game - program through Hopkins - simulation from the ventilator. Learning FEMA's courses were good - interactive. Resources were good - nothing was too long - everything that was bulk reading was broken up which was good.”
	“Liked the different modalities introduced like reading, watching videos, listening to podcasts. Definitely liked the number of resources, alternatives to reading articles which he liked.”
	“Flexibility was nice, my schedule was different than what was reported, that was good to shift stuff around. Being able to pick the time of day.”

## Discussion

We demonstrated that a multimodal asynchronous electronic platform, in conjunction with targeted in-situ activities, leads to a reported understanding of core disaster medicine concepts for pediatric residents. Our curriculum incorporated adult learning theory principles of experiential and self-directed learning and delivers content in accordance with the ACGME’s Systems-Based Practice Core Competency. The modular curriculum incorporates unique in-situ experiences that complement and enhance foundational knowledge and allows for real and simulated application of these knowledge gains [9,10,20].

Trainees provided positive feedback on the flexibility of scheduling in-person experiences to work around the quarantine limitations. The trainees completed the online modules and, when necessary, scheduled the in-person activities at a later time. The COVID-19 pandemic has required considerable flexibility in the provision of education and training, particularly for residents who must self-quarantine if ill or exposed to high-risk patients [21]. The pandemic has also impacted pediatric volumes at most hospitals which has implications for education, making this asynchronous novel curriculum particularly suitable in these settings. Furthermore, providing engaging curricular access during a time of isolation decreases stress and anxiety [22].

The primary limitation of this study is the small sample size as the elective is new and limited to one institution. However, our curriculum can be easily adapted for other residency programs and disasters. Modules 2, 3, 5, and 6 address disaster curricula, not specific to COVID-19, and Modules 1 and 4 could easily be interchanged for other illnesses/disasters. Dissemination of these results may inform other programs trying to implement similar educational interventions. 

## Conclusions

This descriptive study shows how The COVID-19 Elective for Pediatric Residents allows for asynchronous learning, supplemented by relevant in-situ experiences, and provides needed disaster response and resiliency education for pediatric residents. Our results highlight the lack of disaster medicine and hospital response in pediatric residency curriculum, particularly during a time when all physicians need to be knowledgeable and prepared to act on these concepts. This unique curriculum can be modified to any pandemic or global disaster and moreover, to any residency training program. In addition, our continual assessment of the curriculum has enabled real-time modifications and adaptations of course content to learner’s needs. We plan to frequently improve this course and assess long-term knowledge retention. Although further research in teaching pediatric residents disaster response and resilience in a systems-based practice manner is warranted, this well-received curriculum may be a first step in addressing this knowledge gap for pediatric trainees.

## Appendices

Supplemental Content 1. The COVID-19 Elective for Pediatric Residents: Curriculum Content

Module 1

Objectives

This module has three objective and focuses on understanding what coronavirus is, how it presents in pediatric patients, particularly neonates and pregnant patients. By the end of Module #1, the trainee should be able to do the following:

1. Understand the transmission of coronavirus and be able to describe three detection methods.

2. Describe the five key clinical characteristics of COVID-19 in newborns, infants and children.

3. Understand the management of infants born to mothers with COVID-19 in the hospital setting.

*A discussion of Pediatric Multi-system Inflammatory Syndrome (MIS-C) is included in Module 4.

Reading and Tasks

OBJECTIVE 1: OVERVIEW OF CORONAVIRUS (2 hours)

Welcome to OpenWHO, the World Health Organization’s first online, interactive platform. For this portion of Module, you will complete four parts in the course titled “Emerging respiratory viruses, including COVID-19: methods for detection, prevention, response and control.

You will need to create an account (free) and complete the “required” components of the registration. This can be done at the following link:

https://openwho.org/account/new

The four educational modules are as follows and can be accessed through the following link:

https://www.who.int/emergencies/diseases/novel-coronavirus-2019/training/online-training

Module A: Introduction to Emerging respiratory viruses, including COVID-19

Module B: Detecting Emerging respiratory viruses, including COVID-19: Surveillance and Laboratory investigation

Module C: Risk Communication and Community Engagement

Module D: Preventing and Responding to an emerging respiratory virus, including COVID-19

Helpful hints: You can speed up the videos and there are also options to download the videos, pdfs of the slides and transcript of the videos. This may be helpful when completing the quizzes

OBJECTIVE 2: KEY CLINICAL FEATURES OF COVID 19 in PEDIATRICS (2 hours)

Read the following articles and complete the assessment quiz that is located in The Learning Center.

1. Zimmerman et al (2020) “Coronavirus Infection in Children Including COVID-19-An overview of the epidemiology, clinical features, diagnosis, treatment and prevention options in children.”

https://journals.lww.com/pidj/Fulltext/2020/05000/Coronavirus_Infections_in_Children_Including.1.aspx)

2. Dong et al (2020). Epidemiological Characteristics of 2143 Pediatric Patients with COVID.

https://pediatrics.aappublications.org/content/pediatrics/early/2020/03/16/peds.2020-0702.full.pdf

3. Landa et al (2020). Chilblain‐like lesions on feet and hands during the COVID‐19 Pandemic

https://onlinelibrary.wiley.com/doi/10.1111/ijd.14937

4. Molloy and Bearer (2020). COVID-19 in children and altered inflammatory responses

https://www.nature.com/articles/s41390-020-0881-y

OBJECTIVE #3: CLINICAL FEATURES AND MANAGEMENT OF NEONATES BORN TO COVID+ MOTHERS (2.5 hours)

Read the following articles and complete the assessment quiz that is located in The Learning Center.

1. Hong et al (2020). Clinical Characteristics of novel coronovirus disease 2019 in newborns, infants, and Children

https://www.sciencedirect.com/science/article/pii/S1875957220300267?via%3Dihub

2. Puopolo et al (2020). INITIAL GUIDANCE: Management of Infants Born to Mothers with COVID-19

https://downloads.aap.org/AAP/PDF/COVID%2019%20Initial%20Newborn%20Guidance.pdf

Listen to the following podcasts (2 hours):

1. “Covid 19: Pregnancy and Newborns.”

https://www.hippoed.com/peds/rap/episode/bonusshortcovid1/covid19

2. Harvard’s “The Brain Architects Podcast: COVID-19 Special Edition: Creating Communities of Opportunity”

https://developingchild.harvard.edu/resources/the-brain-architects-podcast-covid-19-special-edition-creating-communities-of-opportunity/

3. Dr Mike’s COVID-19: Our New Routine - PediaCast 457

https://www.pediacast.org/covid-19-new-routine-pediacast-457/

Assignments

1. You must obtain 80% on each the four WHO assessments and email your Record of Achievement Certificate to the course director (30 minutes).

2. Complete the 15-question quiz on material from Objectives 2 and 3. You must achieve answer 11/15 questions correctly. One attempt allowed but it is open resource (30 minutes).

Additional Resources

1. CDC (https://www.cdc.gov/coronavirus/2019-ncov/hcp/clinical-guidance-management-patients.html; https://www.cdc.gov/coronavirus/2019-ncov/hcp/pediatric-hcp.html)

2. NEJM (https://www.nejm.org/coronavirus)

3. Podcast (https://covid.hippoed.com/#read)

4. SCCM (SCCM Guidelines COVID19; SCCM-COVID-19-Infographics; SCCM-COVID-19-Infographics2; https://www.sccm.org/disaster)

5. https://hbr.org/2020/03/lessons-from-italys-response-to-coronavirus

6. COVID-19 RASHES (https://www.dermsolutionstx.com/covid?fbclid=IwAR0cHBFxc5OcGB4xqmumheTRw2nKGVoNn8BPhKNB_0YhadHwaP1HY2Iz0FI)

Module 2

Objectives

The goal of this module is to provide the learner with an understanding of the disaster management strategies utilized to identify and manage risks in both the public and private sectors, in addition to creating a resilience foundation that will serve the learner throughout their career.

1. Understand the major components of the resilience life-cycle and the associated techniques used by emergency managers to identify and manage risks for an organization/jurisdiction.

2. Recognize the mechanisms within the preparedness cycle and the methods used to manage risks.

3. Understand the fundamentals of the response system and structure utilized by the public and private sectors to manage incidents, emergencies, crises and disasters.

Reading and Tasks

OBJECTIVE 1: RESILIENCE (1.1 hours) - Understand the major components of the resilience life cycle and the associated techniques used by emergency managers to identify and manage risks for an organization/jurisdiction.

Watch the following two videos:

1. Kevin Thomas’s from Boston University’s talk on Key Components to Healthcare Disaster Management

 https://www.youtube.com/watch?v=ip-mTeGqaqI

2. Know your hospital’s Emergency Manager - The Essential Emergency Manager

 https://www.youtube.com/watch?v=3jXlhPGs0T8&t=7s

OBJECTIVE 2: THE PREPARENESS CYCLE (2.5 hours) - Recognize the key mechanisms within the preparedness cycle and the comprehensive techniques used to manage risks.

Tasks

1. Read this 6-page document from FEMA on  National Preparedness System Description

 https://www.fema.gov/media-library/assets/documents/29361?fromSearch=fromsearch&id=6551

2. Read the following documents on risk identification/assessment:

a. CPG-201: Comprehensive Preparedness Guide 201 

 https://www.fema.gov/media-library/assets/documents/165308

b. Risk Assessments in Healthcare: ASPR TRACIE Evaluation of Hazard Vulnerability Assessment Tools

 https://files.asprtracie.hhs.gov/documents/aspr-tracie-evaluation-of-hva-tools-3-10-17.pdf

3. Validating Capability & Improvement Planning:

Watch this video from Ken Woodall and the Indiana Department of Homeland Security on Homeland Security Exercise and Evaluation Program

 https://www.youtube.com/watch?v=8zPqKBruobg

OBJECTIVE 3: DISASTER RESPONSE (6.5 hours) - Understand the fundamentals of the response system and structure utilized by the public and private sectors to manage incidents, emergencies, crises and disasters and learn how the preparedness and response techniques can utilized to manage operational risks.   Complete the following FEMA courses:

Tasks

1. IS-100.c Introduction to the Incident Command System, ICS 100

 https://training.fema.gov/IS/courseOverview.aspx?code=IS-100.c

2. IS-700.B: An Introduction to the National Incident Management System

 https://training.fema.gov/is/courseoverview.aspx?code=is-700.b

3. IS-1300.d: Introduction to Continuity of Operations

 https://training.fema.gov/IS/courseOverview.aspx?code=IS-1300

Assignments

The Preparedness Cycle

1. Sign up for access to Johns Hopkins PACERSUITE. PACERSUITE has the capability to assess current surge capacity, model plausible disaster scenarios, and forecast the current week’s flu cases. Registration information will need vetted and access may take a few days.

 https://www.pacerapps.org/

a. Review complete a few scenarios, and in less than a page, provide the course Director with your experience using the platform and how tools like PACERSUITE could be utilized to support not only disaster efforts, but operational resilience.

2. Sign up for ASPR TRACIE and review the resources available for future use.

 https://asprtracie.hhs.gov/

a. ASPR TRACIE (short video)

 https://asprtracie.hhs.gov/

b. Review Coronavirus Resources

 https://asprtracie.hhs.gov/COVID-19

Disaster Response: Complete quizzes throughout and at the end of each FEMA IS course.

Additional Resources

1. PrepTalks: Dr. Sheri Fink "Healthcare Emergency Preparedness and Response"

 https://www.youtube.com/watch?v=nKs7MlmEzuY

2. PrepTalks: Dr. Lori Peek "Children and Disasters - Reducing Vulnerability and Building Capacity"

 https://www.youtube.com/watch?v=R7gMetv_9bQ&list=PL720Kw_OojlJiYKDZQwKG7HAgV_qNjbLB&index=31&t=0s

3. IS-366.a: Planning for the Needs of Children in Disasters

 https://training.fema.gov/IS/courseOverview.aspx?code=IS-366.a

4. IS-800.d: National Response Framework, An Introduction

 https://training.fema.gov/IS/courseOverview.aspx?code=IS-800.d

5. Homeland Security Exercise and Evaluation Program

 https://www.fema.gov/media-library/assets/documents/32326

Module 3

Objectives

This module builds upon Module #2 and enables the learner to see how the resilience foundation is applied and utilized at various hospitals, including Nationwide Children’s Hospital (NCH). Additionally, the learner will recognize the realities of developing and enhancing existing resilience programs.

1. Understand the realities and challenges facing Emergency Managers.

2. Review COVID-19 strategies utilized by hospitals of varying size.

3. Identify NCH’s emergency managers in the Emergency Preparedness Department and become familiar with the location of emergency and disaster plans.

4. Obtain hands-on experience with the tactical response teams at NCH

Reading and Tasks

OBJECTIVE 1: ORGANIZATIONAL RESILIENCE: REALITIES AND CHALLENGES (1.4 hours) - Understand the realities and challenges healthcare Emergency Managers face in supporting overall organizational resilience.

Tasks:

1. Listen to EM Weekly’s podcast that discusses what Charles Lane, an emergency manager in one hospital system does in his role. A look In the World of Healthcare Emergency Management

 https://www.spreaker.com/user/sitchradio/ep-82-a-look-in-the-world-of-healthcare-?tab=messages&utm_medium=widget&utm_source=user%3A10696393&utm_term=messages_button

OBJECTIVE 2: OPERATIONS, STRATGIES AND EXPERIENCES (1.5 hours) - Review COVID-19 strategies utilized by hospitals of varying size.

Tasks:

1. View the following webinar on Healthcare System Operations, Strategies and Experiences

 https://register.gotowebinar.com/recording/1619994450926416140

 Must register for instant access

OBJECTIVE 3: KNOW AND UTILIZE (6.5 hours) - Expand on the methodical approach to responding to emergencies and disasters in both the private and public sectors by applying this knowledge to what is being done at NCH. Additionally, identify NCH’s emergency managers located in NCH’s Emergency Preparedness Department and become familiar with the content and location of emergency/disaster plans.

Tasks

1. Complete FEMA’s IS-200.c. Basic Incident Command System for Initial Response

 https://training.fema.gov/IS/courseOverview.aspx?code=IS-200.c

2. Use NCH’s Anchor and find the Emergency Preparedness site

 Review Disaster, Pandemic, & Infectious Control associated plans

OBJECTIVE 4: INTEGRATE AND SUPPORT RESPONSE (2.5-3 days) - Obtain hand-on experience with NCH’s response to the COVID pandemic by integrating and participating in the response.

Tasks:

1. Participate and support Emergency Preparedness for at least a half day.

2. Participate in one half day at the outdoor scheduled testing and laboratory sites at NCH.

3. Participate one half day providing patients with their COVID testing swab results.

Assignments

1. In less than one page, email the course directors with at least three (3) major challenges in Healthcare Emergency Management and how you could support those challenges throughout your career.

2. After reviewing NCH’s emergency plans, identify and contact the Emergency Preparedness Coordinator and provide feedback via email and provide your experience and recommendations relative to the site, resources and documents.    

3. Complete FEMA’s IS-200.c quizzes throughout and at the end of the course

4. Play the online Disaster Triage Game  and save for future use

 http://disastertriagegame.org/index.html

Module 4

Objective

This module focuses on reviewing and rehearsing safe personal protective equipment (PPE) donning and doffing, increasing familiarity with mechanical ventilators, and working through the recognition and stabilization of patients presenting with COVID-19 related Multi-system Inflammatory Syndrome (MIS-C). By the end of Module #4, the trainee should be able to do the following:

1. Demonstrate safe donning and doffing of PPE.

2. Describe five key features of a mechanical ventilator.

3. Describe the clinical features and management of COVID-19-related inflammatory syndrome (MIS-C) in children.

Reading and Tasks

Objective 1: PPE DONNING & DOFFING (6 hours)

Please view the following videos to gain an understanding of appropriate PPE donning and doffing.  The videos reinforce fastidious attention to contamination avoidance while demonstrating variations in PPE.

1. ANCHOR

 http://anchor.columbuschildrens.net/ppe-guidance-1

2. Federal

 https://youtu.be/bG6zISnenPg

 https://www.cdc.gov/hai/pdfs/ppe/ppe-sequence.pdf (summarizing pdf)

3. Hippo ED

 https://www.youtube.com/watch?v=t1lxq2OUy-U

Objective 2: MECHANICAL VENTILATOR KNOWLEDGE & SKILLS (4 hours)

The cognitive component of this objective has 3 modules. The first two combined will take you about an hour. Please allow yourself 4 hours for the third module involving the virtual ventilator simulator.  This is an animated interactive tutorial that includes self-paced exercises and tests. You may need to be on the NCH campus to access the ventilator simulator or use Google Chrome.

1. AHA Training Modules: Non-Invasive Ventilatory Support (introductory)

 https://cpr.heart.org/en/resources/coronavirus-covid19-resources-for-cpr-training/oxygenation-and-ventilation-of-covid-19-patients

2. Dr. Claire Stewart’s PPT presentation on “Management of COVID-19 Related Respiratory Failure.”

3. COVID-19 Airway Management in the Intensive Care Unit; Moynihan & Nagler (a 7-minute video from Open Pediatrics)

 https://www.youtube.com/watch?v=V-FEi9NEYtQ&fbclid=IwAR11ga50Y8YndVBmY84nexh2EkfxxOmUROmlPBQ6Xd9dO0xKf0DUx9uNU3Y

4. Open Pediatrics Virtual Ventilator Simulator

 https://www.openpediatrics.org/assets/simulator/ventilator-simulator

Objective 3: MIS-C RECOGNITION & STABILIZATION (1 hour)

Please read the following information regarding COVID-19-related inflammatory syndrome in children. We encourage you to seek out additional peer-reviewed resources as this topic is evolving.

1. Read the Nationwide Children’s Hospital MISC Clinical Practice Guidelines on Anchor.

2. Feldstein LR et al. Multisystem Inflammatory Syndrome in U.S. Children and Adolescents. N Engl J Med. 2020 Jul 23;383(4):334-346.

3. Kaushik A, Gupta S, Sood M, Sharma S, Verma S. A Systematic Review of Multisystem Inflammatory Syndrome in Children Associated With SARS-CoV-2 Infection. Pediatr Infect Dis J. 2020 Nov;39(11):e340-e346.

4. Sperotto F, Friedman KG, Son MBF, VanderPluym CJ, Newburger JW, Dionne A. Cardiac manifestations in SARS-CoV-2-associated multisystem inflammatory syndrome in children: a comprehensive review and proposed clinical approach. Eur J Pediatr. 2020 Aug 15:1-16.

Simulation!

Please email course directors to confirm your simulation time. (1-3 hours)

1. Demonstrate PPE donning and doffing.

2. Demonstrate 2-person bag-mask ventilation with PPE in the simulation center.

3. Simulate the acute evaluation and initial care of a child presenting with symptoms consistent with COVID-19-related Multisystem Inflammatory Syndrome.

a. Demonstrate knowledge of diagnostic criteria.

b. Describe indications for escalation of therapy.

c. Verbalize clinical orders to implement therapy.

4. Intubate the simulated COVID patient as indicated.

5. Operate and adjust mechanical ventilator for a simulated COVID patient.

6. Delineate the pitfalls and precautions of mechanical ventilation for a simulated COVID patient.

Assignments

1. Complete the Open Pediatrics Ventilator Simulator Assessments.

2. Participate in a simulation and demonstrate knowledge and skills as noted above.

Optional Activities

Optional Activities 1. Top 5 Things All Healthcare Providers Should Know (a pdf from the AHA)

 https://cpr.heart.org/-/media/cpr-files/resources/covid-19-resources-for-cpr-training/oxygenation-and-ventilation-of-covid-19-patients/ds16042-top-5-cpr-covid_fin.pdf?la=en

2. Critical Care for the on-ICU Clinician:

 https://covid19.sccm.org/nonicu.htm 

Module 5

Objective

This module focuses on the mental health ramifications of COVID-19 and similar disasters on the children, healthcare providers and the community at large. This module also lists various mental health support resources. By the end of this module, the trainee should be able to:

1. Describe three way in which global disasters and pandemics impact community mental health.

2. Describe three ways in which global disasters and pandemics impact physician mental health.

3. Identify resources available in Central Ohio to provide mental health support for the community.

4. Access resources available at NCH that support health care providers with regards to mental health concerns.

Reading and Tasks

OBJECTIVE 1: GLOBAL MENTAL HEALTH IMPACT OF THE COVID-19 PANDEMIC (4-5 hours)

Although it is only starting to be explored, it is clear that COVID-19 and the responses to it have caused major disruptions to the lives of billions of people around the world. It is also evident that the mental health impact of this virus will be broad and long-lived. Below are some readings that will provide you with an overview of this topic. We also encourage you to find additional relevant literature, as this topic will be evolving rapidly for months (and possibly years).

Readings

1. Interagency Standing Committee Briefing note on addressing mental health and psychosocial aspects of COVID-19 Outbreak- Version1.

 https://interagencystandingcommittee.org/system/files/2020-03/MHPSS%20COVID19%20Briefing%20Note%202%20March%202020-English.pdf

2. Caring for Patients’ Mental Well-Being During Coronavirus and Other Emerging Infectious Diseases: A Guide for Clinicians

 https://www.cstsonline.org/assets/media/documents/CSTS_FS_Caring_for_Patients_Mental_WellBeing_during_Coronavirus.pdf

3. COVID-19 pandemic: impact on psychiatric care in the United States.

 https://www.ncbi.nlm.nih.gov/pmc/articles/PMC7200362/

OBJECTIVE 2: THE PANDEMIC EFFECT ON MENTAL HEALTH OF MEDICAL PROVIDERS (1-2 hours)

It is well documented that health care providers may experience psychological trauma from their experiences at work, especially while caring for patients with COVID-19. During times where stressors are severe and prolonged, the risk of mental health deterioration for providers increases. Resources are available, but may not be widely known. Furthermore, providers may not take advantage of the resources that exist. We have provided readings below and again, this is an evolving topic, so we encourage you to find additional resources.

Reading/Video

1. Sustaining the Well-Being of Healthcare Personnel during Coronavirus and other Infectious Disease Outbreaks

 https://www.cstsonline.org/assets/media/documents/CSTS_FS_Sustaining_Well_Being_Healthcare_Personnel_during.pdf

2. Innovative Approaches to Enhancing Resident Resilience, Well-being and Engagement

 https://www.youtube.com/watch?v=L5Xq7JsbRSg&feature=youtu.be

OBJECTIVE #3: LOCAL COMMUNITY RESOURCES FOR PEDIATRIC MENTAL HEALTH  (1-2 hours)

There has been an increasing emphasis on pediatric mental health over recent years leading to the development of some pediatric-specific mental health resources. However, awareness and access are still considerably less than they should be, and as demonstrated above, mental health issues are becoming even more pressing during the COVID-19 pandemic. Thus, it is important to know what resources exist in order to be able to direct patients and families to what can best serve them.

Readings:

1. Review the community resources available in Franklin County. Many of these can be found on the NCH website:

 https://www.nationwidechildrens.org/giving/on-our-sleeves/find-help/state-resources/ohio/columbus-resources

2. Listen, Protect, Connect for Children and Parents. May be found at

 https://www.fema.gov/media-library/assets/documents/132712

Task:

1.   Watch a live or recorded webinar about pediatric mental health. They can be found here:

 https://www.nationwidechildrens.org/specialties/behavioral-health/for-providers/webinar-series

OBJECTIVE #4: LOCAL MENTAL HEALTH RESOURCES FOR PROVIDERS (2 hours)

While health care can be a stressful profession at any time, this has been accentuated by the exigencies of the COVID-19 pandemic. This is particularly true in heavily impacted areas (eg: New York and Italy) where providers were in the position of having to ration care. Once again, awareness of available resources is critical in ensuring that all affected providers have access to the aid they need to take care of themselves.

Readings:

1. Familiarize yourself with the You Matter program: YOU Matter Second Victim Team - intended to support clinicians after an acute traumatic stressor.

 http://anchor.columbuschildrens’net/you-matter-second-victim-team

2. The STAR program at NCH provides support to clinicians experiencing an acute traumatic stressor: STAR Program at NCH

 http://anchor.columbuschildrens.net/stress-trauma-and-resilience-program

3. The Federal Healthcare Resilience Task Force created self-care guidelines for frontline providers:

 https://www.911.gov/pdf/Burnout_Self-Care_COVID-19_Exposure_for_First_Responders.pdf

Assignments

1. Spend ½ days shadowing the PEEC clinicians at the Behavioral Health Pavilion. Try to focus on understanding the reasons pediatric patients present to the BHP and how COVID-19 is brought up and addressed during the visits. If you select this option, contact course director to schedule the shadowing experience. This can take place of #3 below.

2. Create an account and complete the 15 minute video on self-care and psychological first aid at Ohio Train:

 https://www.train.org/odh/course/1090639/

This video provides the healthcare responder with just-in-time training on responder stress during COVID-19, and provides stress management strategies that should be implemented now to protect our healthcare workforce.

3. Please select one of the following to participate in and email the course directors once you have completed the activity.

A. Participate in a You Matter Zoom Meeting.

OR

B. Meet with a You Matter clinician to help gain an understanding of their role.

Final Assignment

There are four options to complete the assessment for this module. Please complete one of them and email it to the course directors.

Option 1: Complete Psychological First Aid training at https://learn.nctsn.org/enrol/index.php?id=38. You will need to create an account and the training takes about 6 hours. This is for learners particularly interested in participating in the mental health response to a crisis. Proof of completion will be accepted as the assessment for this module.

Option 2: Design a mental health crisis response plan for either patients or providers in a particular context (for example, for patients in an NCH primary care clinic). As a guideline, this should be about 1 page, but use what space you need.

Option 3: Select an available mental health resource and design an informative means of increasing awareness of it. This may take the form of a poster, press release, podcast, etc. Be creative.

Option 4: Write 1-2 paragraphs on approaches to self-care during the SARS-CoV-2 pandemic.

Additional Resources

1. Franklin County Youth Psychiatric Crisis Line - (614) 722-1800

2. Skills for Psychological Recovery Field Operations Guide

 https://www.ptsd.va.gov/professional/treat/type/SPR/SPR_Manual.pdf

3. PsySTART Mental Health Triage

 https://www.youtube.com/watch?v=4ZTmUlpxeBM&feature=emb_logo

4. Listen-Protect-Connect Model of Psychological First Aid

 https://www.youtube.com/watch?v=a8p-4a80NAM

Module 6

Objective

By the end of this module, the trainee should be able to:

1. List the sciences essential to public health practice.

2. State the limitations of testing capabilities locally and nationally.

3. Identify the steps that local public health offices take in closing down and reopening businesses during a pandemic.

4. Compare the four components of contact tracing.

Reading and Tasks

OBJECTIVE 1: PUBLIC HEALTH 101 (5 hours)

According to the Institute of Medicine, public health is a coordinated effort at the local, state, and federal levels whose mission is fulfilling society’s interest in assuring conditions in which people can be healthy. The CDC has a designed an introduction/refresher six-part series on public health that provides an overview of the fundamental scientific components of public health practice. We ask that you complete the following sections, though feel free to take the others.

https://www.cdc.gov/publichealth101/index.html

 Introduction to Public Health (approx. 60 minutes)

 Introduction to Epidemiology (approx. 60 minutes)

 Introduction to Prevention Effectiveness (approx. 45 minutes)

 Introduction to Public Health Informatics (approx. 65 minutes) [OPTIONAL]

 Introduction to Public Health Surveillance (approx. 45 minutes)

 Introduction to Public Health Laboratories (approx. 36 minutes)

Please also read the following:

1. Always The Bridesmaid, Public Health Rarely Spotlighted Until It’s Too Late.

 https://khn.org/news/always-the-bridesmaid-public-health-rarely-spotlighted-until-its-too-late/

2. Optimizing the Health and Well-Being of the Nation’s Children.

 https://pediatrics.aappublications.org/content/141/2/e20173848

3. What is Child Public Health?

 https://www.sciencedirect.com/science/article/pii/S0957583904001150

OBJECTIVE 2: NATIONAL AND LOCAL TESTING LIMITATIONS

1. Explore this webpage:

 https://www.cdc.gov/cpr/whatwedo/phep.htm

2. Listen to this 22-minute podcast on “How to Track an Epidemic”:

 https://sph.umich.edu/podcast/season2/disease-detectives.html

OBJECTIVE 3: CLOSING AND REOPENING THE STATE AND COUNTRY

The ramifications of “closing” and “reopening” the state and nation blends the health and economic costs of our society. Tough decisions to declare stay at home/shelter in place orders and the closures of schools and businesses and public places are weighed against healthcare resources/ the Public’s health. These decisions are most felt at the local level. We are asking you to read the following RAND blog post.

https://www.rand.org/blog/2020/05/reopening-america-the-health-and-economic-tradeoffs.html

Keep these questions and points in mind when you go to Columbus Public Health (CPH) for your 4 hours Immersive Experience. During your time at CPH you will meet with local public health officials who will guide you through the roles and responsibilities the local public health department undertakes, especially during pandemics. Please pay special attention to inspections (schools, daycares, etc.) who rely on insight from pediatricians when making decisions. This will be a great networking opportunity for you.

OBJECTIVE 4: CONTACT TRACING

1. COVID-19 Policy Tracker:

 https://www.multistate.us/pages/covid-19-policy-tracker?utm_campaign=phpartners&utm_medium=email&utm_source=govdelivery

 https://preventepidemics.org/wp-content/uploads/2020/04/COV020_WhenHowLoosenFaucet_v4.pdf

2. Contact Tracing

 https://www.cdc.gov/coronavirus/2019-ncov/php/open-america/contact-tracing.html

 Please watch the approx. 3-minute video.

 https://www.cdc.gov/coronavirus/2019-ncov/downloads/php/principles-contact-tracing-booklet.pdf

 https://www.cdc.gov/coronavirus/2019-ncov/downloads/php/prelim-eval-criteria-digital-contact-tracing.pdf

Listen to this 11-minute podcast on Contact Tracing:

 https://sph.umich.edu/podcast/coronavirus/public-health-surveillance.html

Read this article:

 https://jamanetwork.com/journals/jama/fullarticle/2762689

Assignments

1. Each module in the CDC’s Public Health 101 has knowledge assessment questions embedded in the presentations/slide decks. Please complete these. 

2. Please complete Public Health 101 prior to your Immersive Experience at Columbus Public Health. Doing so will make you familiar with the concepts, components of public health, and functions of the health department. This will enrich your experience and allow you to more accurately articulate the intersection of the role of the pediatrician, the local public health department, and the health of children. This immersive experience with Columbus Public Health is aimed to foster greater understanding of how the local public health system works and improved engagement through networking with public health officials.

You will be scheduled by the course directors to take part in a visit to the Columbus Public Health Department.

After you have completed your Immersive Experience at Columbus Public Health, please spend 10 minutes writing a reflection of how you think your role as a pediatrician intersects with the work of Columbus Public Health. Once complete, send your writing to the course director.

Optional Activities

1. Overview of Public Health Reading

 https://www.cpha.ca/sites/default/files/uploads/policy/ph-framework/phcf_e.pdf

2. Beating Back the Devil: On the Front Lines with the Disease Detectives of the Epidemic Intelligence Service by Maryn McKenna (2004)

 https://www.amazon.com/Beating-Back-Devil-Maryn-McKenna/dp/1439123101

3. Listen to this 8 minute podcast on talking to kids about COVID-19.

 https://sph.umich.edu/podcast/coronavirus/how-to-talk-to-kids-about-coronavirus.html

4. Listen to this 11 minute podcast about the well-being of kids and COVID-19.

 https://www.stitcher.com/podcast/centreinfection/infectious-questions

5. Listen to this 14 minute podcast about Connecting with Youth during COVID-19.

 https://americanhealth.libsyn.com/keeping-youth-connected-during-coronavirus

Supplemental Content 1 (cont.): References for The COVID-19 Elective for Pediatric Residents Curricular Content

Module 1:

World Health Organization “Coronovirus disease (COVID 19) training: Online Training.” Retrieved from https://www.who.int/emergencies/diseases/novel-coronavirus-2019/training/online-training. Accessed May 2020.

Zimmermann P, Curtis N. Coronavirus Infections in Children Including COVID-19. 2020. The Pediatric Infectious Disease Journal. 39(5): 355-368. 

Dong Y, Mo X, Hu Y, et al. Epidemiological characteristics of 2143 pediatric patients with 2019 coronavirus disease in China. Pediatrics. 2020; doi: 10.1542/peds.2020-0702

Landa N, Mendieta-Eckert M, Fonda-Pascual P, Aguierre T. Chilblain-like lesions on feet and hands during COVID 19 Pandemic. International Journal of Dermatology. 2020;59(6): 739-743.

Molloy, E.J., Bearer, C.F. COVID-19 in children and altered inflammatory responses. 2020. Pediatr Res . https://doi.org/10.1038/s41390-020-0881-y

Hong H, Wang Y, Chung HT, Chen CJ. Clinical characteristics of novel coronavirus disease 2019 (COVID-19) in newborns, infants and children. Pediatr Neonatol. 2020;61(2):131‐132. doi:10.1016/j.pedneo.2020.03.001

Behar S and Parga-Belinkie J. “COVID 19: Pregnancy and Newborns.” Retrieved from https://www.hippoed.com/peds/rap/episode/bonusshortcovid1/covid19. Accessed May 2020.

Pfitzer S and Williams D. “The Brain Architects Podcast: COVID 19 Special Edition: Creating Communities of Opportunity. Retrieved from https://developingchild.harvard.edu/resources/the-brain-architects-podcast-covid-19-special-edition-creating-communities-of-opportunity/. Accessed May 2020.

Hiltebeitel C. “Podcast: COVID 19 Information for Parents and Children. Retrieved from https://developingchild.harvard.edu/resources/the-brain-architects-podcast-covid-19-special-edition-creating-communities-of-opportunity/. Accessed May 2020.

Patrick M. Pediacast 457. “COVID 19: Our New Routine.” Retrieved from https://www.pediacast.org/covid-19-new-routine-pediacast-457/. Accessed May 2020.

Module 2:

Thomas K. “10 Keys to Healthcare Emergency Planning.” Retrieved from https://www.pediacast.org/covid-19-new-routine-pediacast-457/. Accessed May 2020.

Black Swan Emergency Manager “The Essential Emergency Manager.” Retrieved from https://www.pediacast.org/covid-19-new-routine-pediacast-457/. Accessed May 2020.

Department of Homeland Security. “National Preparedness System Description.” 2011. Retrieved from https://www.fema.gov/media-library/assets/documents/29361?fromSearch=fromsearch&id=6551. Accessed May 2020.

Department of Homeland Security. “Comprehensive Preparedness Guide (CPG) 201: Threat and Hazard Identification and Risk Assessment (THIRA) and Stakeholder Preparedness Review (SPR) Guide.” 2018. Retrieved from https://www.fema.gov/media-library-data/1527613746699-fa31d9ade55988da1293192f1b18f4e3/CPG201Final20180525_508c.pdf. Accessed May 2020.

“ASPR TRACIE Evaluation of Hazard Vulnerability Assessment Tools.” October 2018. Retrieved from https://files.asprtracie.hhs.gov/documents/aspr-tracie-evaluation-of-hva-tools-3-10-17.pdf. Accessed May 2020.

Woodall K. “Homeland Security Exercise and Evaluation Program Overview.” Retrieved from https://www.youtube.com/watch?v=8zPqKBruobg. Accessed May 2020.

FEMA Emergency Management Institute. “IS-100.C: Introduction to the Incident Command System, ICS 100.” June 2018. Retrieved from https://training.fema.gov/is/courseoverview.aspx?code=IS-100.c. Accessed May 2020.

FEMA Emergency Management Institute. “IS-700.B: An Introduction to the National Incident Management System.” June 2018. Retrieved from https://training.fema.gov/is/courseoverview.aspx?code=is-700.b. Accessed May 2020.

FEMA Emergency Management Institute. “S-1300: Introduction to Continuity of Operations.” Nov 2019. Retrieved from https://training.fema.gov/is/courseoverview.aspx?code=IS-1300. Accessed May 2020.

Johns Hopkins “PACERSUITE.” Retrieved from https://www.pacerapps.org. Accessed May 2020.

“Welcome to ASPR TRACIE.” Retrieved from https://asprtracie.hhs.gov. Accessed May 2020.

“ASPR TRACIE Novel Coronavirus Resources.” Retrieved from https://asprtracie.hhs.gov/COVID-19. Accessed May 2020.

Fink S. “PrepTalks: Healthcare Emergency Preparedness and Response.” Retrieved from https://www.youtube.com/watch?v=nKs7MlmEzuY. Accessed May 2020.

Peek L. “PrepTalks: Children and Disasters - Reducing Vulnerability and Building Capacity.” Retrieved from https://www.youtube.com/watch?v=I2a0DezEA7Y&list=PL720Kw_OojlJiYKDZQwKG7HAgV_qNjbLB&index=2. Accessed May 2020.

FEMA Emergency Management Institute. “IS-366.A: Planning for the Needs of Children in Disasters.” Retrieved from https://training.fema.gov/is/courseoverview.aspx?code=IS-366.a. Accessed May 2020.

FEMA Emergency Management Institute.” IS-800.D: National Response Framework, An Introduction.” Retrieved from https://training.fema.gov/is/courseoverview.aspx?code=IS-800.d. Accessed May 2020.

“Homeland Security Exercise and Evaluation Program.” January 2020. Retrieved from https://www.fema.gov/media-library-data/1582669862650-94efb02c8373e28cadf57413ef293ac6/Homeland-Security-Exercise-and-Evaluation-Program-Doctrine-2020-Revision-2-2-25.pdf. Accessed May 2020.

Module 3:

DeVoe T. EM Weekly’s Podcast. “EP 82 A look In The World of Healthcare Emergency Management.” Retrieved from https://www.spreaker.com/user/sitchradio/ep-82-a-look-in-the-world-of-healthcare-?tab=messages&utm_medium=widget&utm_source=user%3A10696393&utm_term=messages_button. Accessed May 2020.

FEMA Emergency Management Institute. “IS-200.C: Basic Incident Command System for Initial Response.” March 2019. Retrieved from https://training.fema.gov/is/courseoverview.aspx?code=IS-200.c. Accessed May 2020.

“Disaster Triage Game.” Retrieved from http://disastertriagegame.org/index.html. Accessed May 2020.

Module 4:

“NETEC: Personal Protective Equipment for COVID-19.” Feb 2020. Retrieved from https://www.youtube.com/watch?v=bG6zISnenPg&feature=youtu.be. Accessed May 2020.

Center for Disease Control and Prevention “Sequence for Putting On and Removing Personal Protective Equipment.” Retrieved from https://www.cdc.gov/hai/pdfs/ppe/ppe-sequence.pdf. Accessed May 2020.

Hippo Education. “PPE Donning and Doffing: CDC Sequence for COVID 19.” March 2020. Retrieved from https://www.youtube.com/watch?v=t1lxq2OUy-U. Accessed May 2020.

American Heart Association “Oxygenation and Ventilation of COVID-19 Patients.” Retrieved from https://cpr.heart.org/en/resources/coronavirus-covid19-resources-for-cpr-training/oxygenation-and-ventilation-of-covid-19-patients. Accessed May 2020.

Moynihan K and Nagler J. “COVID-19 Airway Management in the Intensive Care Unit.” April 2020. Retrieved from https://www.youtube.com/watch?v=V-FEi9NEYtQ&fbclid=IwAR11ga50Y8YndVBmY84nexh2EkfxxOmUROmlPBQ6Xd9dO0xKf0DUx9uNU3Y. Accessed May 2020.

Open Pediatrics “Ventilator Simulator.” Retrieved from https://www.openpediatrics.org/assets/simulator/ventilator-simulator. Accessed May 2020.

Feldstein LR et al. Multisystem Inflammatory Syndrome in U.S. Children and Adolescents. N Engl J Med. 2020 Jul 23;383(4):334-346. doi: 10.1056/NEJMoa2021680. Epub 2020 Jun 29. PMID: 32598831; PMCID: PMC7346765.

Kaushik A, Gupta S, Sood M, Sharma S, Verma S. A Systematic Review of Multisystem Inflammatory Syndrome in Children Associated With SARS-CoV-2 Infection. Pediatr Infect Dis J. 2020 Nov;39(11):e340-e346.

Sperotto F, Friedman KG, Son MBF, VanderPluym CJ, Newburger JW, Dionne A. Cardiac manifestations in SARS-CoV-2-associated multisystem inflammatory syndrome in children: a comprehensive review and proposed clinical approach. Eur J Pediatr. 2020 Aug 15:1-16. doi: 10.1007/s00431-020-03766-6. Epub ahead of print.

American Heart Association “5 Things Every Healthcare Provider Should Know About CPR for COVID-19 Patients.” Retrieved from https://cpr.heart.org/-/media/cpr-files/resources/covid-19-resources-for-cpr-training/oxygenation-and-ventilation-of-covid-19-patients/ds16042-top-5-cpr-covid_fin.pdf?la=en. Accessed May 2020.

Society of Critical Care Medicine “COVID 19 Resources for non-ICU clinicians.” Retrieved from https://covid19.sccm.org/nonicu/. Accessed May 2020.

Module 5:

“Interim Briefing Note Addressing Mental Health and Psychosocial Aspects of COVID-19 Outbreak (developed by the IASC’s Reference Group on Mental Health and Psychosocial Support).” March 2020. Retrieved from https://interagencystandingcommittee.org/iasc-reference-group-mental-health-and-psychosocial-support-emergency-settings/interim-briefing. Accessed May 2020.

Uniformed Services University Center for the Study of Traumatic Stress.”Caring for Patients’ Mental Well-Being During Coronavirus and Other Emerging Infectious Diseases: A Guide for Clinicians.” Retrieved from https://www.cstsonline.org/assets/media/documents/CSTS_FS_Caring_for_Patients_Mental_WellBeing_during_Coronavirus.pdf. Accessed May 2020.

Bojdani E, Rajagopalan A, Chen A et al. COVID-19 pandemic: impact on psychiatric care in the United States. Psychiatry Res. 2020; 289: epub ahead of print.  PMID 32413707

Uniformed Services University Center for the Study of Traumatic Stress. “Sustaining the Well-Being of Healthcare Personnel during Coronavirus and other Infectious Disease Outbreaks.” Retrieved from https://www.cstsonline.org/assets/media/documents/CSTS_FS_Sustaining_Well_Being_Healthcare_Personnel_during.pdf. Accessed May 2020.

Zhu J, Sun L, Zhang L, et al.  Prevalence and influencing factors of anxiety and depression symptoms in the first-line medical staff fighting against COVID-19 in Gansu. Front Psychiatry. 2020; 29: ecollection.  PMID 32411034

On Our Sleeves: The Movement to Transform Children’s Mental Health. “Columbus Resources.” Retrieved from https://www.nationwidechildrens.org/giving/on-our-sleeves/find-help/state-resources/ohio/columbus-resources. Accessed May 2020.

FEMA. “Listen, Protect and Connect (LPC) Psychological First Aid System. Retrieved from https://www.fema.gov/media-library-data/1499092051917-115ad4c12a44f04a93b4a37c17e99211/PFA(1).pdf. Accessed May 2020.

“Behavioral Health Webinar Series.” Retrieved from https://www.nationwidechildrens.org/specialties/behavioral-health/for-providers/webinar-series. Accessed May 2020.

“Federal Healthcare Resilience Task Force EMS/Prehospital Team BURNOUT, SELF-CARE & COVID-19 EXPOSURE FOR FIRST RESPONDERS.” Retrieved from https://www.911.gov/pdf/Burnout_Self-Care_COVID-19_Exposure_for_First_Responders.pdf. Accessed May 2020.

Ohio Department of Health, Ohio TRAIN. “Self-Care and Applying Psychological First Aid for COVID-19 Responders” Retrieved from https://www.train.org/odh/course/1090639/. Accessed May 2020.

The National Child Traumatic Stress Network. “Psychological First Aid Training.” Retrieved from https://learn.nctsn.org/login/index.php. Accessed May 2020.

“Skills for Psychological Recovery: Field Operations Guide.” Retrieved from https://www.ptsd.va.gov/professional/treat/type/SPR/SPR_Manual.pdf. Accessed May 2020.

“PsySTART Mental Health Triage for the District of Columbia: DC Fire and EMS.” December 2015. Retrieved from https://www.youtube.com/watch?v=4ZTmUlpxeBM&feature=emb_logo. Accessed May 2020.

“Webinar #6: Psychological First Aid - Listen Protect Connect/Model and Teach.” May 2020. Retrieved from https://www.youtube.com/watch?v=a8p-4a80NAM. Accessed May 2020.

Module 6:

Centers for Disease Control and Prevention “Public Health 101 Series.” Retrieved from https://www.cdc.gov/publichealth101/index.html. Accessed May 2020.

Rovner J. “Always The Bridesmaid, Public Health Rarely Spotlighted Until It’s Too Late.” May 2020. Retrieved from https://khn.org/news/always-the-bridesmaid-public-health-rarely-spotlighted-until-its-too-late/. Accessed May 2020.

Kuo A, Thomas PA, Chilton LA, Mascola L. Council on Community Pediatrics and Section on Epidemiology, Public Health and Evidence. Pediatricians and Public Health: Optimizing the Health and Well-Being of the Nation’s Children. Pediatrics. 2018;  141 (2): e20173848; DOI: https://doi.org/10.1542/peds.2017-3848

Cresswell T. What is Child Public Health. Current Paediatrics. 2014; 14(7): 612-618.

“CDC’s Public Health Emergency Preparedness Program: Every Response is Local.” Retrieved from https://www.cdc.gov/cpr/whatwedo/phep.htm. Accessed May 2020.

University of Michigan School of Public Health. “Disease Detectives: How to Track an Epidemic.” Retrieved from https://sph.umich.edu/podcast/season2/disease-detectives.html. Accessed May 2020.

“COVID-19 Policy Tracker.” Retrieved from https://www.multistate.us/pages/covid-19-policy-tracker?utm_campaign=phpartners&utm_medium=email&utm_source=govdelivery. Accessed May 2020.

Centers for Disease Control and Prevention. “Contact TracingGet and Keep America Open: Supporting states, tribes, localities, and territories.” Retrieved from https://www.cdc.gov/coronavirus/2019-ncov/php/open-america/contact-tracing-resources.html?CDC_AA_refVal=https%3A%2F%2Fwww.cdc.gov%2Fcoronavirus%2F2019-ncov%2Fphp%2Fopen-america%2Fcontact-tracing.html. Accessed May 2020.

Centers for Disease Control and Prevention “COVID-19 Case Investigation and Contact Tracing for Health Departments.” Retrieved from https://www.cdc.gov/coronavirus/2019-ncov/downloads/php/principles-contact-tracing-booklet.pdf. Accessed May 2020.

Centers for Disease Control and Prevention “Preliminary Criteria for the Evaluation of Digital Contact Tracing Tools for COVID 19. Retrieved from https://www.cdc.gov/coronavirus/2019-ncov/downloads/php/prelim-eval-criteria-digital-contact-tracing.pdf. Accessed May 2020.

University of Michigan School of Public Health “Public Health Surveillance: Immunity, Testing, and Contact Tracing.” Retrieved from https://sph.umich.edu/podcast/coronavirus/public-health-surveillance.html. Accessed May 2020.

Wang CJ, Ng CY, Brook RH. Response to COVID-19 in Taiwan: Big Data Analytics, New Technology, and Proactive Testing. JAMA. 2020;323(14):1341-1342. doi:10.1001/jama.2020.3151

Canadian Public Health Association Working Paper “Public Health: A Conceptual Framework.” Retrieved from https://www.cpha.ca/sites/default/files/uploads/policy/ph-framework/phcf_e.pdf. Accessed May 2020.

McKenna M. “Beating Back the Devil.” 2004. Free Press, NY.

University of Michigan School of Public Health “How to talk to kids about coronavirus.” Retrieved from https://sph.umich.edu/podcast/coronavirus/how-to-talk-to-kids-about-coronavirus.html. Accessed May 2020.

Wierbowski A and Tipples G. Podcast “EP. 27: Serology and COVID-19 (2019-nCoV, Pt 12).” Retrieved from https://www.stitcher.com/podcast/centreinfection/infectious-questions. Accessed May 2020.

Johns Hopkins University’s The American Health Podcast: “The Coronavirus Crisis: Keeping Youth Connected.” May 2020. Retrieved from https://americanhealth.libsyn.com/keeping-youth-connected-during-coronavirus. Accessed May 2020.

Supplemental Content 2. Survey and Interview Questions for the COVID-19 Elective for Pediatric Residents

Please answer the following questions using the prompt:

1. “I believe the Pandemic Elective enhanced my knowledge on …”

a. Current evidence-based literature for COVID-19

b. Disaster preparedness

c. Hospital response to pandemics

d. Taking care of the critically ill child/adult during a pandemic

e. Mental health concerns during a pandemic

f. Public health practices during a pandemic

(Respondents answered using the following options: Strongly Disagree, Disagree, Neither Agree nor Disagree, Agree, Strongly Agree)

2. “I believe the self-assessments adequately assessed my knowledge of …”

a. Current evidence-based literature for COVID-19

b. Disaster preparedness

c. Hospital response to pandemics

d. Taking care of the critically ill child/adult during a pandemic

e. Mental health concerns during a pandemic

f. Public health practices during a pandemic

(Respondents answered using the following options: Strongly Disagree, Disagree, Neither Agree nor Disagree, Agree, Strongly Agree)

3. “The additional resources and/or in-person sessions were meaningful and enhanced my experience on …”

a. Current evidence-based literature for COVID-19

b. Disaster preparedness

c. Hospital response to pandemics

d. Taking care of the critically ill child/adult during a pandemic

e. Mental health concerns during a pandemic

f. Public health practices during a pandemic

(Respondents answered using the following options: Strongly Disagree, Disagree, Neither Agree nor Disagree, Agree, Strongly Agree)

4. “The independent learning time was adequate to complete the required tasks/assignments for the module: …”

a. Current evidence-based literature for COVID-19

b. Disaster preparedness

c. Hospital response to pandemics

d. Taking care of the critically ill child/adult during a pandemic

e. Mental health concerns during a pandemic

f. Public health practices during a pandemic

(Respondents answered using the following options: Strongly Disagree, Disagree, Neither Agree nor Disagree, Agree, Strongly Agree)

5. Please answer the following questions on your beliefs about the elective.

a. The Pandemic Elective enhanced my knowledge overall about pandemics.

b. The learning center online platform was easy to navigate.

c. Information presented in this curriculum was relevant to my clinical practice.

d.  Information presented in this curriculum will change my clinical practice.

(Respondents answered using the following options: Strongly Disagree, Disagree, Neither Agree nor Disagree, Agree, Strongly Agree)

6. Please list two strengths of this elective. (Forced free response)

7. Please list two ways in which this elective can be improved. (Forced free response)

Please answer these basic demographic questions.

8. What gender do you identify with?

Female

Male

Prefer not to answer

9. What is your training program at Nationwide Children’s Hospital?

Pediatric Residency

Medicine - Pediatric Residency

Other combined Pediatric Residency

Subspecialty Fellowship

Comment:

10. What was your training level while completing the curriculum?

PGY-1

PGY-2

PGY-3

PGY-4

PGY-5

11. To which rotation were you assigned while completing the elective?

Inpatient

Outpatient

Research

Elective
